# Glucocorticoids Suppress Renal Cell Carcinoma Progression by Enhancing Na,K-ATPase Beta-1 Subunit Expression

**DOI:** 10.1371/journal.pone.0122442

**Published:** 2015-04-02

**Authors:** Thu P. Huynh, Sonali P. Barwe, Seung J. Lee, Ryan McSpadden, Omar E. Franco, Simon W. Hayward, Robert Damoiseaux, Stephen S. Grubbs, Nicholas J. Petrelli, Ayyappan K. Rajasekaran

**Affiliations:** 1 Molecular Biology Institute, University of California Los Angeles, Los Angeles, California, United States of America; 2 California NanoSystems Institute, University of California Los Angeles, Los Angeles, California, United States of America; 3 Nemours Center for Childhood Cancer Research, A. I. DuPont Hospital for Children, Wilmington, Delaware, United States of America; 4 Department of Urologic Surgery, Vanderbilt University, Nashville, Tennessee, United States of America; 5 Helen F. Graham Cancer Center, Christiana Care Health System, Newark, Delaware, United States of America; 6 Department of Biological Sciences, University of Delaware, Newark, Delaware, United States of America; 7 Therapy Architects, 2700 Silverside Road, Wilmington, Delaware, United States of America; Seoul National University, REPUBLIC OF KOREA

## Abstract

Glucocorticoids are commonly used as palliative or chemotherapeutic clinical agents for treatment of a variety of cancers. Although steroid treatment is beneficial, the mechanisms by which steroids improve outcome in cancer patients are not well understood. Na,K-ATPase beta-subunit isoform 1 (NaK-β_1_) is a cell-cell adhesion molecule, and its expression is down-regulated in cancer cells undergoing epithelial-to mesenchymal-transition (EMT), a key event associated with cancer progression to metastatic disease. In this study, we performed high-throughput screening to identify small molecules that could up-regulate NaK-β_1_ expression in cancer cells. Compounds related to the glucocorticoids were identified as drug candidates enhancing NaK-β_1_ expression. Of these compounds, triamcinolone, dexamethasone, and fluorometholone were validated to increase NaK-β_1_ expression at the cell surface, enhance cell-cell adhesion, attenuate motility and invasiveness and induce mesenchymal to epithelial like transition of renal cell carcinoma (RCC) cells *in vitro*. Treatment of NaK-β_1_ knockdown cells with these drug candidates confirmed that these compounds mediate their effects through up-regulating NaK-β_1_. Furthermore, we demonstrated that these compounds attenuate tumor growth in subcutaneous RCC xenografts and reduce local invasiveness in orthotopically-implanted tumors. Our results strongly indicate that the addition of glucocorticoids in the treatment of RCC may improve outcome for RCC patients by augmenting NaK-β_1_ cell-cell adhesion function.

## Introduction

Kidney cancer is among the most frequent cancers diagnosed [1]. Renal cell carcinoma (RCC) consists of three main histologic types: clear cell, papillary, and chromophobe [2]. Clear cell is the most clinically aggressive and represents the largest group of RCCs [2, 3]. Early stage RCC is resolved by partial or radical nephrectomy if the malignancy is localized, while advanced stages of disease are managed by cytoreductive therapy combined with drugs that target specific oncogenic pathways, such as the mammalian target of rapamycin (mTOR) [4]. The management of advanced RCC remains a significant challenge to clinicians and new therapeutic options are needed for patients with advanced disease.

The Na,K-ATPase is a ubiquitously expressed enzyme that derives energy from ATP to couple the translocation of three Na^+^ out and two K^+^ into the cell, generating a transmembrane sodium gradient that drives the uptake and release of diverse solutes by epithelial cells. The functional Na,K-ATPase consists of α- and β-subunits. The Na,K-ATPase α-subunit (NaK-α) is responsible for the catalytic function of the enzyme. The Na,K-ATPase β-subunit (NaK-β) associates with NaK-α in the endoplasmic reticulum to protect it from degradation and ensures the correct assembly and insertion of the mature enzyme into the plasma membrane. An accessory γ–subunit belonging to the FXYD family of proteins exhibits tissue specific expression and modulates Na,K-ATPase enzyme activity [5]. Of the four α- and three β-subunit isoforms known, the α_1_-subunit (NaK-α_1_) and β_1_-subunit (NaK-β_1_) are prominently expressed in most tissues, particularly in the kidney [6].

Studies from our laboratory and others have demonstrated the loss of NaK-β_1_ expression at the mRNA and protein level, and in some cases, its mislocalization has been noted in several cancers [7–9]. Furthermore, the marked reduction of NaK-β_1_ expression is associated with a higher risk for bladder cancer recurrence [8]. These initial studies implicated a potential role for NaK-β_1_ in attenuating tumor progression and prompted functional studies at the molecular level.

Recently, it has become evident that the NaK-β_1_ serves additional functions beyond its role in ion transport. NaK-β_1_ functions as a cell adhesion molecule and plays a critical role in the development of cell-cell contacts [10–16]. Re-expression of NaK-β_1_ along with the cell-cell adhesion molecule, E-cadherin, in kidney carcinoma cells abrogated cell motility and invasiveness in a synergistic manner and induced mesenchymal-to-epithelial transition (MET) [17]. TGF-β_1_ induced epithelial-to-mesenchymal transition (EMT) in kidney proximal tubule epithelial cells is associated with the reduced expression of both NaK-β_1_ and E-cadherin, and over-expression of NaK-β_1_ alone delayed the progression of EMT [18]. Anchorage independent growth is a hallmark of malignant transformation, and tumor suppressors block this feature [19]. Restoration of NaK-β_1_ expression in kidney carcinoma cells blocked anchorage- independent growth and reduced tumorigenicity in immuno-compromised mice [12]. More recently we showed that the NaK-β_1_ promoter is silenced by hyper methylation in RCC [20] like many other tumor suppressors [21]. These studies indicate that NaK-β_1_ has a tumor suppressor function in renal epithelial cells.

Given its plethora of roles in suppressing the tumor phenotype, enhancement of NaK-β_1_ expression in renal cancer cells may represent a novel mechanism for RCC treatment. Our approach was to identify compounds that augment NaK-β_1_ expression and examine their ability to prevent or delay the motility and invasiveness of kidney cancer cells. We focused on kidney cancer, since many of the previous studies exploring the anti-tumor roles of NaK-β_1_ have been characterized in kidney cells [6, 15, 16, 22]. We performed a high-throughput screen (HTS) using a cell-based gene reporter assay and identified several members of the glucocorticoid class of compounds. Here, we report that triamcinolone (TRIAM), dexamethasone (DEX), and fluorometholone (FLUOR) enhance cell-cell aggregation and reduce both the motility and invasiveness of kidney cancer cells *in vitro* in a NaK-β_1_ dependent manner. Additionally, we found that the drugs were effective in reducing tumor growth in a subcutaneous xenograft mouse model and the local invasiveness of orthotopically implanted kidney tumor cells in severe combined immunodeficient (SCID) mice. These studies support the use of glucocorticoids to attenuate progression of renal neoplasms through up-regulation of NaK-β_1_.

## Materials and Methods

### Cell lines and reagents

HeLa and Caki-1 cells from ATCC were maintained as described by the supplier (ATCC Rockville, MD). UMRC6 cells were from Dr. Michael I. Lerman (National Cancer Institute, Bethesda, MD) and maintained in RPMI with 10% FBS, 1 mM glutamine, 100 U/mL penicillin, and 100 μg/mL streptomycin [23]. DEX (Tocris Bioscience, Ellisville, MI), TRIAM and FLUOR (Sigma-Aldrich, St Louis, MO) were prepared in dimethyl sulfoxide (DMSO) (EMD Chemicals, Gibbstown, NJ) at 10,000-fold stock solution. Cells were serum starved prior to treatment and routinely treated with 100 nM or 10 μM of compound in serum free medium or medium containing charcoal-stripped FBS (Invitrogen, Carlsbad, CA) for 24 hr. For immunostaining, cells were treated with 10 μM for 3 days before fixation.

### shRNA and transfections

The full-length NaK-β_1_ promoter fused to firefly luciferase described previously [9] was co-transfected with pBABE-puromycin into HeLa cells and single clones were selected after puromycin treatment. Positive clones were confirmed by luciferase assay after addition of DEX. shRNA against human NaK-β_1_ (shRNA-β) targets the sequence 5’-GTGATGCTGCTCACCATCA-3’ [18], was cloned into pSilencer (Applied Biosystems, Austin, TX), and transfected into Caki-1 as described previously [24]. For transfection of ptd-Tomato-N1 (Clontech, Mountain View, CA), nucleofector technology was used (Lonza, Walkersville, MD). Single cells expressing red fluorescent protein were picked after selection with G418 to establish stable cell lines.

### Screening protocol

Cells were seeded in phenol-red free DMEM (Invitrogen, Carlsbad, CA) in white 384-well plates (ThermoFisher, Hudson, NH). Small molecule libraries were obtained from Biomol International LP (Plymouth Meeting, PA), MicroSource Inc. (Ann Arbor, MI), Prestwick Chemical (Washington, DC), Asinex (Moscow, Russia), and ChemBridge (San Diego, CA). Compounds were dissolved in DMSO and transferred into assay plates using a Biomek FX (Beckman Coulter, Brea, CA) equipped with a 384-pin tool (V&P Scientific, San Diego, CA). The final compound concentration was 10 μM except the Biomol library, which was used according to the manufacturer’s recommendation. Luciferase activity was assessed after 24 hr. Steady-lite (Perkin-Elmer, Waltham, MA) was added and luciferase activity was measured with a Victor3 plate reader (Perkin-Elmer). The hit cutoff was selected as 80% or more of the activity induced by DEX.

### Antibodies

Na,K-ATPase α_1_- (M7-PB-E9) and β_1_-subunit (M17-P5-F11) antibodies have been previously well-characterized [25, 26]. Actin antibody was obtained from Sigma. N-Cadherin was from BD Biosciences (Franklin Lakes, NJ).

### Quantitative PCR

RNA isolated with RNAqueous Kit (Ambion, Austin, TX) was reverse transcribed using the High-Capacity cDNA Archive Kit (Applied Biosystems, Foster City, CA). Taqman probes specific for human NaK-α_1_, NaK-β_1_, and hypoxanthine phosphoribosyl transferase (HPRT) were from Applied Biosystems. Q-PCR was performed with a 7900HT Fast Real-Time PCR system (Applied Biosystems). Samples were assayed in triplicate and normalized to HPRT. All data represent the mean of three to four independent experiments ± standard deviation.

### Immunoblotting

Cells were washed with PBS and lysed in lysis buffer (20 mM Tris-HCl, pH 7.4, 100 mM NaCl, 1% Triton X-100, 1 mM EDTA, 1 mM EGTA, 1 mM sodium glycerolphosphate, 1 mM sodium orthovanadate, 1 mM PMSF, and 5 μg/ml each of antipain, leupeptin, and pepstatin). After sonication and clarification, the supernatants were collected and protein estimated (Bio-Rad, Hercules, CA). Equal amounts of total protein were separated by SDS-PAGE and transferred to nitrocellulose membrane (Schleicher & Schuell, Keene, NH). Blocking occurred in 5% nonfat dry milk in PBS with 0.1% Tween 20 (PBS-T). Primary antibodies were diluted in blocking solution and membranes were incubated overnight at 4°C. Blots were washed with PBS-T and developed with the enhanced chemiluminescence western lightning system (Perkin-Elmer, Waltham, MA).

### Cell surface biotinylation assay

Cells were washed in cold PBS containing 1 mM MgCl_2_ and 0.1 mM CaCl_2_ (PBS-CM). EZ-Link Sulfo-NHS-LC-Biotin (ThermoFisher) freshly prepared in DMSO was diluted into TEA buffer (150 mM NaCl, 10 mM triethanolamine pH 9, 1 mM CaCl_2_, and 1 mM MgCl_2_) at 4 mg/mL and reacted with the cells on ice. After PBS-CM washes, excess biotin was quenched with 50 mM NH_4_Cl in PBS-CM. Cells were lysed in lysis buffer. Samples were sonicated, protein estimated, pre-cleared with Protein A agarose beads, and equal amounts of protein were incubated with streptavidin beads (ThermoFisher) at 4°C for 12 hrs. Beads were washed three times with cold lysis buffer and processed for immunoblotting as described above.

### Cell-cell aggregation assay

Cells pre-treated for 24 hr with 100 nM GCs were washed in PBS and trypsinized. After neutralization, 10 mM MgCl_2_ and 10 μg/mL of DNase I was added to the cell suspension and incubated at 37°C for 15 min. The single cell suspension was prepared in SMEM (Invitrogen), and cells were seeded onto 12-well dishes coated with 1% (w/v) agarose and incubated on a shaker at 100 rpm. After 1 hr, cells were fixed with 4% paraformaldehyde and single cells were counted with a hemocytometer. Aggregation index was calculated using the formula, (*N*0 − *N*1)/*N*0, where *N*1 is the single cell number after the incubation time of 1 hr and *N*0 is the cell number at the initiation of incubation.

### Wound healing assay

This was performed as described previously [14]. Cells were pre-treated with GCs for 24 hrs, and a uniform cell-free area was created and inspected regularly. At each time point, nine phase contrast images were acquired with a Leica DMIL microscope (Leica Microsystems). The distance between the two opposing edges was measured. Values represent the mean±SE of three independent experiments with 27 measurements taken for each condition at each time point in each experiment.

### Transwell invasion assay

The Transwell invasion assay was performed as described previously [14]. Transwell inserts (8 μm pore) (Becton-Dickinson, Franklin Lakes, NJ) were coated with rat-tail collagen type I (BD Biosciences, Franklin Lakes, NJ). Cells were re-suspended in medium containing DMSO or drugs and seeded onto the insert. After treatment, the insert was washed, and a cotton swab dipped in trypsin was used to remove cells from the top of the insert. Cells attached to the underside were fixed and stained in crystal violet. The insert was thoroughly washed, and cell-associated crystal violet was eluted with 10% acetic acid. The quantity of cells that migrated through correlated to the absorbance of eluted crystal violet measured at 595 nm. Migration of drug-treated samples was expressed as a percent of migration compared to control vehicle-treated cells.

### Immunofluorescence staining

Caki-1 cells incubated with control vehicle or GCs were fixed with 4% paraformaldehyde and then permeabilized with 0.3% TritonX-100 in PBS. Non-specific binding was blocked with goat and donkey serum and then incubated overnight at 4°C in a humidified chamber with diluted antibodies. N-Cadherin antibody was used at a dilution of 1:200 and Alexa488-conjugated phalloidin (Invitrogen, Carlsbad, CA) for 1:1,000. After washing with PBS, cells were incubated with FITC or Texas red conjugated secondary antibodies (Jackson Immunoresearch, West Grove, PA) for an hour. Coverslips were mounted with Prolong Gold antifade solution (Invitrogen, Carlsbad, CA) which contains DAPI. Images were obtained by using a confocal microscope (SP8, Leica Microsystems) with a 40x objective lens.

### Animal studies

All experiments involving animals were performed in accordance with protocols reviewed and approved by the Nemours Institutional Animal Care and Use Committee. Four-week old male hairless SCID mice were obtained to conduct *in vivo* studies (Charles River Laboratory, Malvern, PA). Mice were housed in pathogen-free conditions with a 12 hr light/12 hr dark schedule and fed with autoclaved standard chow and water *ad libitum*. Mice were injected subcutaneously with 2.5 x 10^6^ cells/mouse in 0.1 mL of sterile PBS. Tumor dimensions were monitored by measurement with a caliper and by fluorescent imaging with KODAK *in vivo* Multispectral imaging system (Carestream Health, Rochester, NY). Orthotopic transplantation was performed as described previously [27]. Caki-1 cells expressing ptd-Tomato were trypsinized during mid-log phase of growth, pelleted, and re-suspended in rat-tail collagen prepared as described previously [28] at a density of 5,000 cells/μL. Cells were spotted onto 10 cm dishes. After the spots gelled to form cell plugs, they were overlaid with growth medium and incubated at 37°C overnight. The collagen plugs were then grafted under the renal capsule of one of the kidneys of SCID mice. Five days after graft insertion, mice were randomly assigned into 4 groups with 10 mice per group. Each group was injected i.p. with 5 mg/kg weight of TRIAM, DEX, or FLUOR in 100 μL PBS containing 2% DMSO every other day. At 38 days post-surgery, mice were sacrificed. Kidneys were removed for imaging and processed for histology.

### Immunohistochemistry

Kidneys were fixed in formalin, embedded in paraffin, and 4–5 μm thick sections were mounted on slides. Slides were air dried, baked at 55°C, placed into Reveal solution (Biocare, Concord, CA), and heated at 125°C for 30 sec in a decloaking chamber (Biocare) followed by dipping in Hot Rinse solution (Biocare) at 90°C. Water was slowly added to the slides and rinsed with PBS. Endogenous peroxidase was quenched with 1% H_2_O_2_. Slides were blocked with 4% FBS in PBS. DsRed antibody (Clontech) was added to the slides and incubated overnight at 4°C in a humidified chamber. Unbound antibody was gently rinsed off slides with PBS. The MACH4 Universal Detection System (Biocare) was used to develop the slides following manufacturers’ instructions. Slides were then stained with hematoxylin and eosin. Slides were dehydrated in 100% ethanol and xylene and mounted with EcoMount. Slides were viewed under bright-field illumination with a Leica DMIL microscope (Leica Microsystems).

### Analysis and scoring

Immunohistochemical staining for DsRed was used to determine localization of tumor tissue. The entire tumor boundary was scanned for invasive patches within the renal cortex. An invasive patch is defined as an area of DsRed positive cells penetrating the capsule wall. The average number of invasive patches in each treatment category was determined and plotted.

### Statistical analysis

All data are expressed as means±SEs. A Student’s *t*-test was used to examine relationships between the control and drug treated groups. *P*-values of less than 0.05 were considered statistically significant. All experiments, except the *in vivo* studies, were repeated at least 3 times.

## Results

### High-throughput screen to identify small molecules that stimulate Na,K-ATPase β_1_-subunit promoter activity

We utilized a human cervical cancer cell line (HeLa) widely used in high-throughput screening (HTS) studies [29, 30] to create a cell-based gene reporter assay to identify compounds that enhance NaK-β_1_ gene expression. HeLa cells stably harboring a construct containing the firefly luciferase gene under the control of the NaK-β_1_ promoter (H1141β-Luc) were used to probe the small molecule library. Prior to screening, the assay conditions were optimized and validated to ensure that the assay can distinguish between true sample signals versus background noise. We achieved a Z’-factor of 0.5, which is considered reliable for such assays [31]. DMSO and DEX was included in each assay plate as negative and positive controls, respectively. An overwhelming majority of the compounds identified in the HTS were glucocorticoids (GCs) (S1 Table). We focused on GCs, since many of them are already FDA approved therapeutics for a broad array of pathologic conditions.

**Fig 1:**
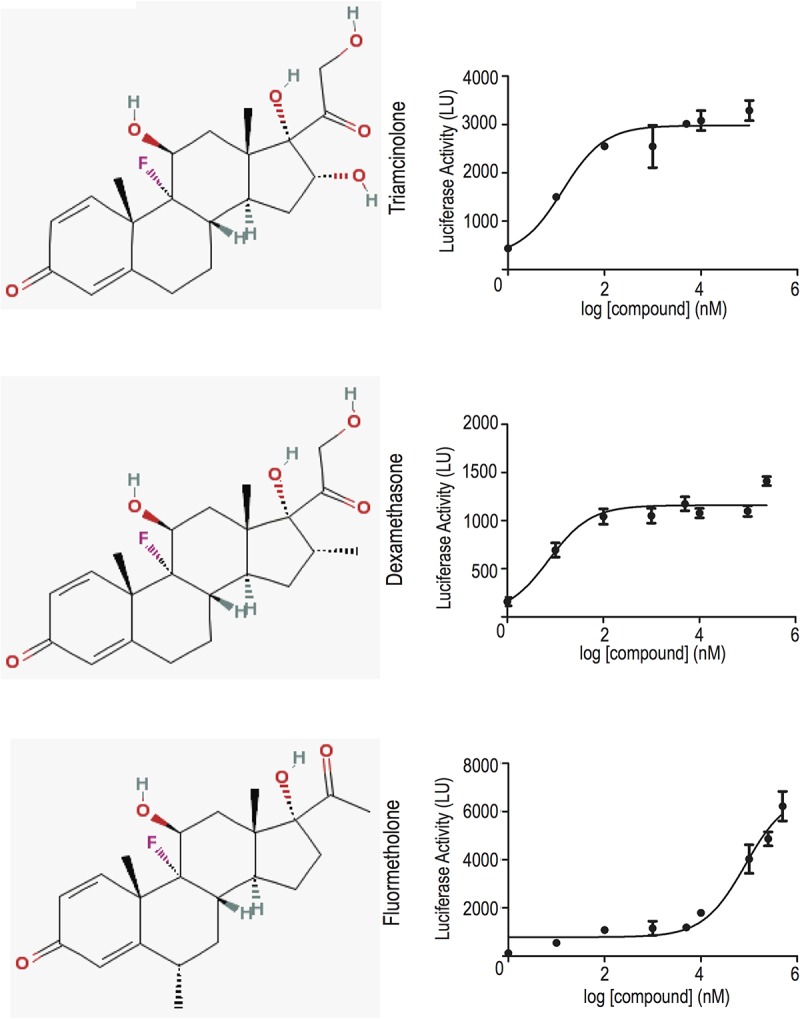
Steroidal drugs enhance NaK-β_1_ promoter activity. Compounds were tested at 1 nM to 250 μM and the luciferase activity was measured in light units (LU). There is a clear dose-dependent response in TRIAM, DEX, and FLUOR exposed HeLa cells.

**Fig 2:**
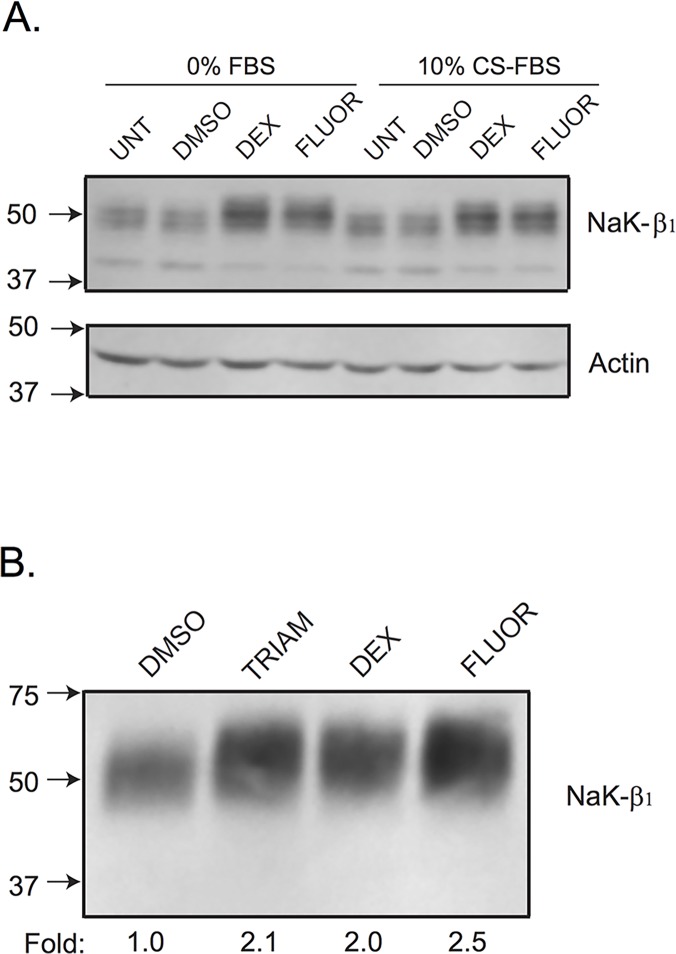
Validation of the small molecule candidates in HeLa cells. (A) Immunoblot confirmation of NaK-β_1_ up-regulation in the presence or absence of drugs. HeLa cells were treated with DEX or FLUOR in the presence or absence of charcoal-stripped serum. The compounds produced a similar degree of NaK-β_1_ induction regardless of serum content. Additionally, the control vehicle, DMSO, did not alter NaK-β_1_ expression compared to untreated cells. (B) HeLa cells were treated with 10 μM TRIAM, DEX, or FLUOR for 24 hr and a cell surface biotinylation assay was performed to elucidate the amount of NaK-β_1_ at the cell surface. A representative blot from three independent experiments confirm the localization of NaK-β_1_ at the cell exterior with compound treatment compared to DMSO treated cells.

Triamcinolone (TRIAM) and fluorometholone (FLUOR) were further studied based on their solubility and strength of induction of NaK-β_1_ promoter activity compared to DEX (S1 Table). Compounds identified by HTS were verified by dosing studies, which demonstrated a dose-dependent increase in NaK-β_1_ promoter activity with increasing drug concentrations in HeLa cells (Fig 1). Next, we assessed if NaK-β_1_ protein levels were augmented with drug treatment by immunoblot analysis. With the addition of drugs, NaK-β_1_ expression was elevated in serum free as well as in charcoal-stripped serum (CS) (Fig 2A). Furthermore, the increased NaK-β_1_ was properly targeted to the cell membrane as assessed by a cell surface biotinylation assay (Fig 2B).

### Validation of GCs mediated induction of NaK-β_1_ expression in renal clear cell carcinoma cells

**Fig 3:**
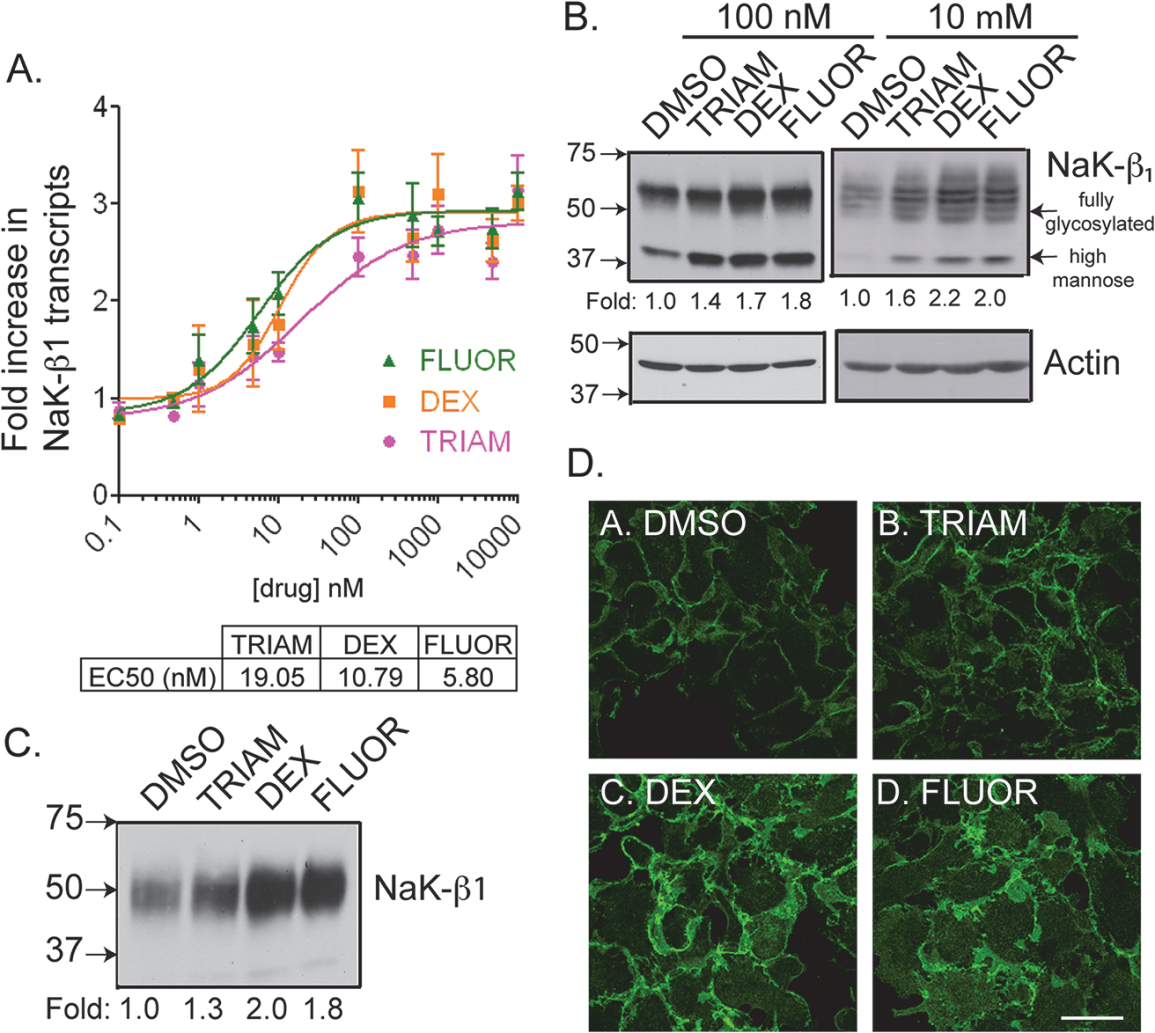
GC compounds elevate NaK-β_1_ expression in kidney cancer cells. (A) NaK-β_1_ transcript levels were determined by Q-PCR and expressed as a fold change over DMSO-treated cells. Drugs induced a dose-dependent increase in NaK-β_1_ transcripts. Data represents the mean±SE of 3 independent experiments performed in quadruplicates. (B) Immunoblot analysis showing NaK-β_1_ levels in Caki-1 cells treated with DMSO or GCs. Actin confirmed equal loading of protein. (C) All three drugs induced increased NaK-β_1_ at the plasma membrane at 100 nM and 10 μM in a cell-surface biotinylation assay. (D) Immunostaining of NaK-β_1_ (green) in Caki-1 drug treated cells imaged by confocal microscopy. TRIAM (panel B), DEX (panel C), and FLUOR (panel D) exhibited more intense NaK-β_1_ staining both intracellularly and at the cell surface compared to DMSO (panel A) treated cells. Note the thicker staining along the cell-cell contacts. Scale bar represents 20 μm.

Caki-1, a renal clear cell carcinoma (RCC) cell line that has been extensively used for studies on RCC [32, 33], was used to characterize the function of the drugs in RCC. We determined whether the identified compounds exert their effects at the transcriptional level. Quantitative-PCR analysis showed that GCs increased NaK-β_1_ mRNA levels in a dose-dependent manner. TRIAM, DEX, and FLUOR enhanced NaK-β_1_ gene expression with EC50s of 19.05, 10.79, and 5.80 nM, respectively (Fig 3A). 100 nM and 10 μM concentrations were used for further studies because these concentrations are within the range applied clinically [34, 35].

Na,K-ATPase protein levels in DMSO- and GC-treated cells were analyzed by immunoblotting. TRIAM, DEX, and FLUOR enhanced NaK-β_1_ expression in Caki-1 cells (Fig 3B). Cell surface biotinylation of Caki-1 cells treated with TRIAM, DEX, and FLUOR revealed 1.3±0.1, 2.0±0.1, and 1.8±0.1-fold enhanced NaK-β_1_ expression at the plasma membrane, respectively, compared to DMSO-treated cells (Fig 3C). The enhanced cell surface expression of NaK-β_1_ was also confirmed by immunofluorescence (Fig 3D). In summary, GCs induced *NaK-β*_*1*_ expression both at the transcript and protein levels in Caki-1 cells.

### GCs increased cell-cell adhesion and reduced the motility and invasiveness of kidney cancer cells

**Fig 4:**
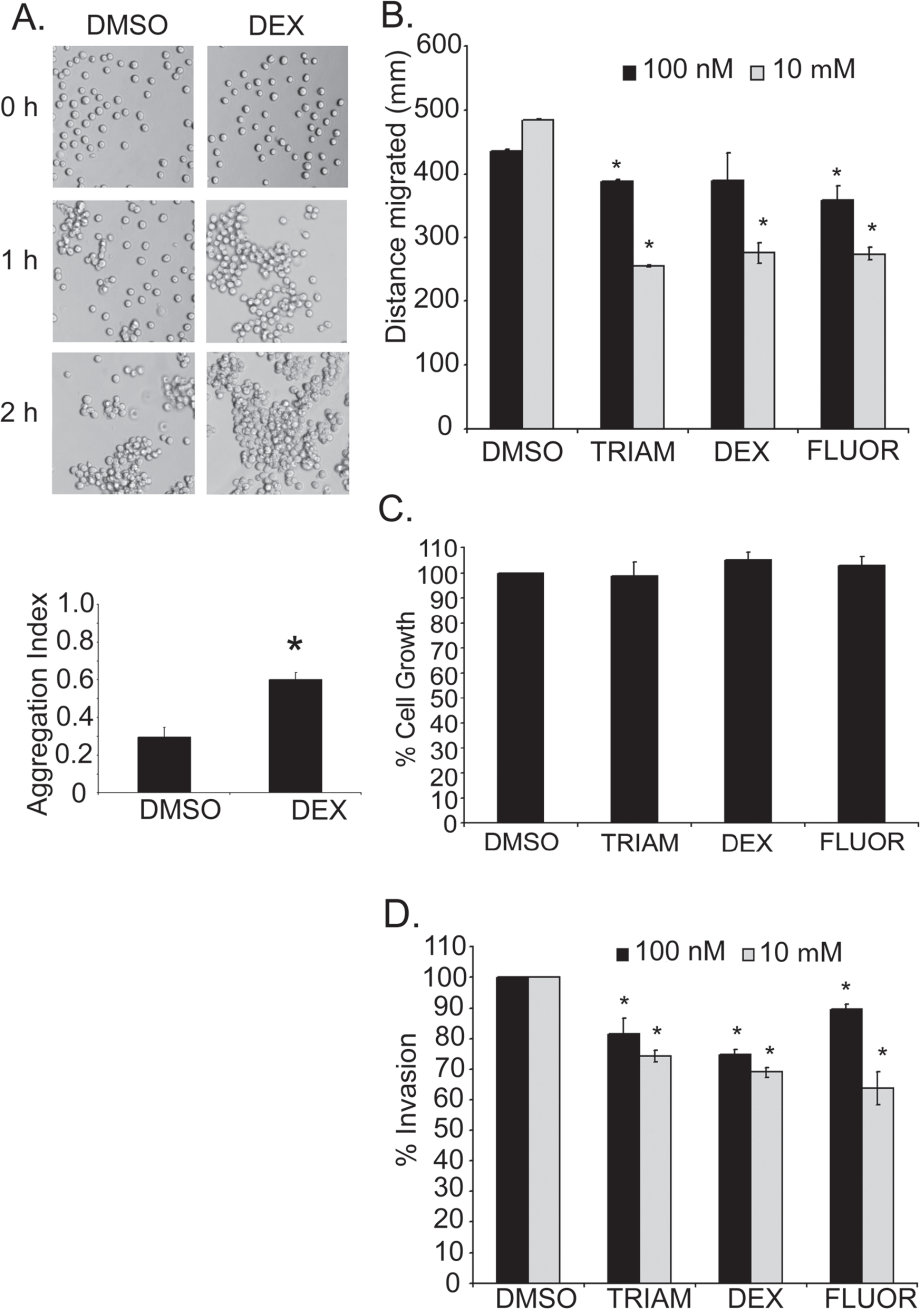
GCs enhanced cell-cell aggregation and reduced the motility and invasiveness of kidney cancer cells. (A) Phase contrast images of a cell-cell aggregation assay showing the ability of GCs to enhance cohesion of cells. The graph represents the mean of three independent experiments performed in quadruplicate wells ±SE. (B) The distance migrated by cells pre-treated with DMSO or GCs in a wound healing assay after 24 hr. The data represent the mean±SE of three independent experiments. Asterisk indicates p-value <0.05 by Student’s *t*-test comparing DMSO versus drug treatment. (C) The reduction of Caki-1 migration by drug treatment was not a result of changes in cell proliferation as shown by an MTT assay. This was performed in three independent experiments with 12 wells per condition. The graph is from a single representative experiment with bars representing the mean±SE. (D) A Transwell invasion assay showing the invasive capacity of Caki-1 cells through collagen coated inserts at 24 hr. Data represent means±SE of three independent experiments performed in duplicate wells. Asterisk indicates significant p-value < 0.05 by Student’s *t*-test.

Previously, our laboratory and others have shown that *NaK-β*_*1*_ mediates cell-cell adhesion [11, 12, 15–17, 22, 36]. We tested the ability of GCs to alter cellular adhesion *in vitro*. After 24 hr, DEX-treated cells displayed more cell-cell cohesion, produced larger cell clusters, and revealed a two-fold increase in the aggregation index compared to DMSO-treated cells (p-value = 0.0086) (Fig 4A).

Several reports have demonstrated that *NaK-β*_*1*_ reduces tumor cell migration and invasiveness [14, 22, 37]. The migration of Caki-1 cells treated with 100 nM TRIAM, DEX, or FLUOR was reduced compared to DMSO-treated controls (389.2±1.0 μm, p-value = 0.000031; 404.4±28.2 μm, p-value = 0.326; and 363.8±14.4 μm, p-value = 0.0077; respectively, compared to 436.0±2.0 μm) (Fig 4B). The effect of 10 μM TRIAM, DEX, and FLUOR was greater (255.5±1.97, 275.9±16.1, 274.4±9.7 μm, respectively, compared to 458.8±1.6 μm) (Fig 4B). A cell viability assay showed that treatment of cells for 24 hr with the drugs did not alter the viability compared to DMSO controls (Fig 4C). Thus, the difference in migration distance between DMSO- and GC-treated cells is not a consequence of changes in cell growth.

**Fig 5:**
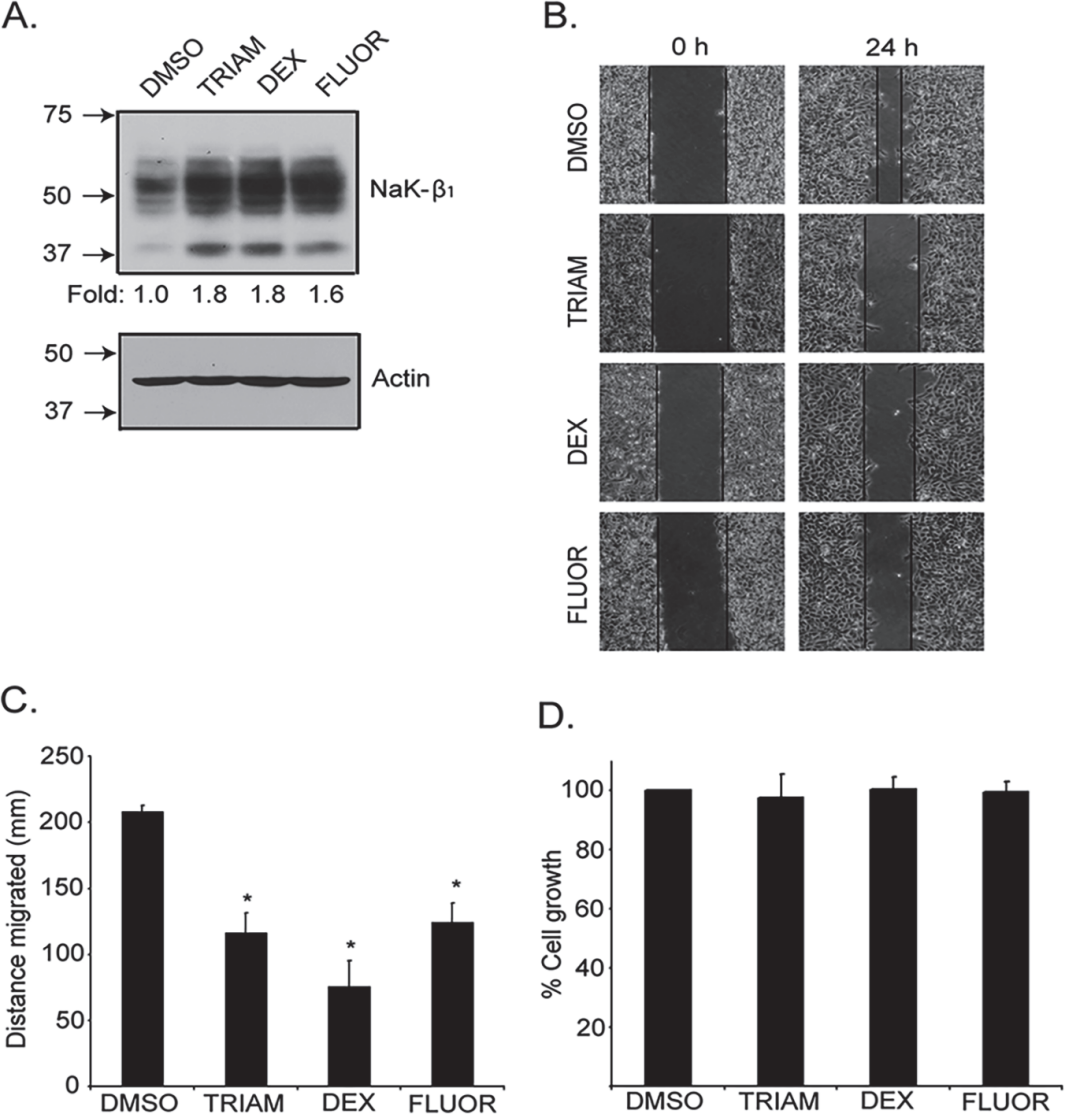
The anti-tumor effects were reproduced in UMRC6 cells. (A) The small molecule drugs were effective in up-regulating NaK-β_1_ protein levels in the human renal clear cell carcinoma line, UMRC6, as observed in the immunoblot. (B) Additionally, the drugs were efficacious in reducing the motility of UMRC6 cells as demonstrated by the wound healing assay. Drug treated cells migrated a shorter distance. (C) Quantification of the wound healing indicates that the compounds reduced the distance migrated. (D) The attenuation in motility was not due to changes in the rate of cell growth, since DMSO and drug treated cells grew comparably as revealed by a MTT assay.

A Transwell invasion assay with TRIAM, DEX, and FLUOR (100 nM) treatment reduced the percentage of invading Caki-1 cells to 81.5±5.2% (p-value = 0.069), 74.8±1.7% (p-value = 0.0047), and 89.7±1.5% (p-value = 0.020) respectively, compared to DMSO controls (Fig 4D). At 10 μM TRIAM, DEX and FLUOR the invasiveness was further reduced (74.3±1.9%, p-value = 0.0027; 69.0±1.7%, p-value = 0.000053; and 63.8±5.4%, p-value = 0.00018, respectively). The ability of the drugs to enhance *NaK-β*_*1*_ *and reduce motility was* confirmed in another human RCC cell line, UMRC6. Treatment of UMRC6 cells increased protein expression of Na,K-β_1_ by 1.6–1.8-fold (Fig 5A). This was accompanied by a reduction in cell migration after 24 hr drug treatment (Fig 5B and 5C). The reduction of cell migration was not due to alterations in UMRC6 growth, since a MTT assay showed that cell growth was similar in control vehicle and drug treated cells over this time period (Fig 5D). Taken together, these results confirm that GCs enhance NaK-β_1_ expression and reduce cancer cell motility and invasion in kidney cancer cells.

### GCs function through NaK-β_1_ to enhance cell-cell aggregation and reduce invasion

**Fig 6:**
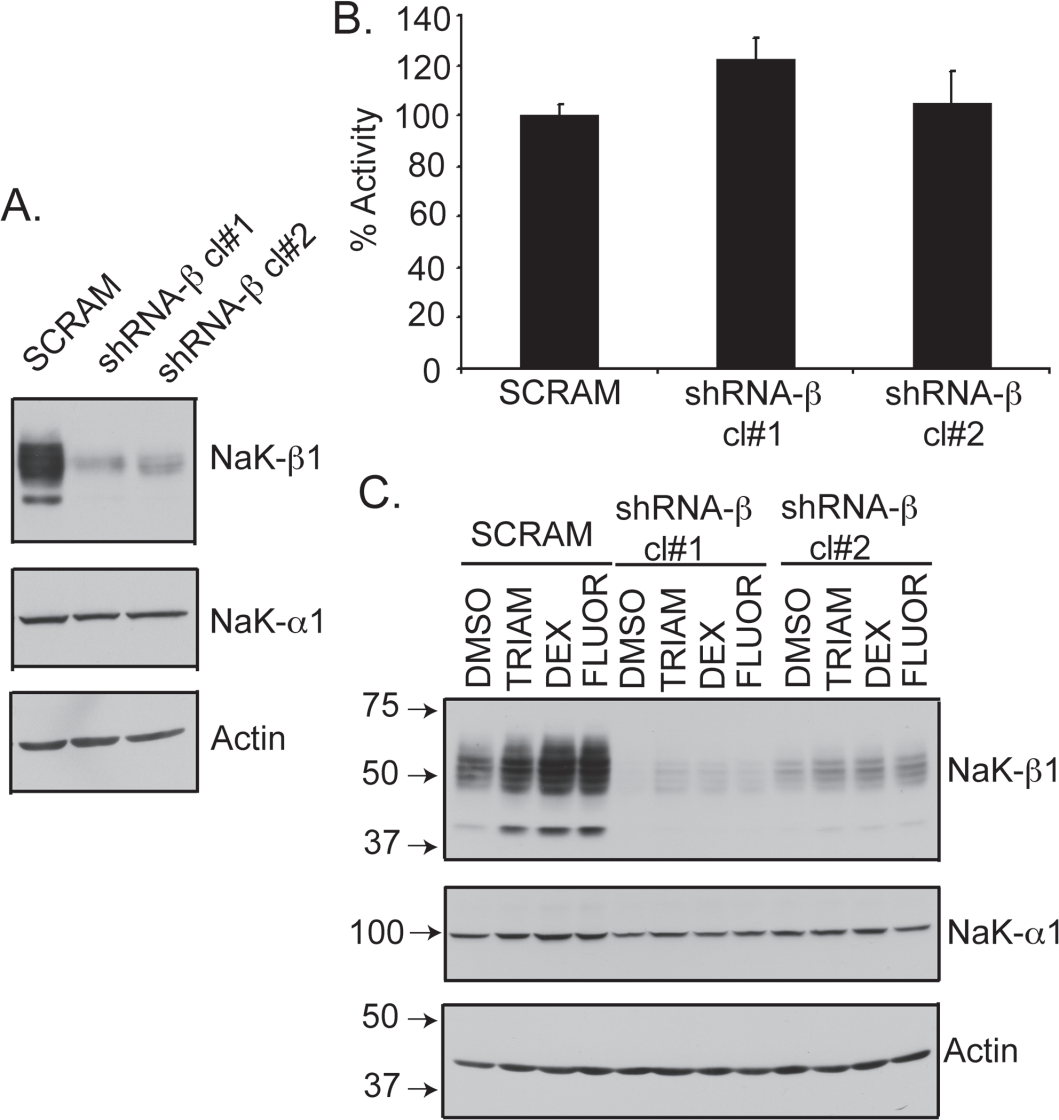
Characterization of shRNA-β clones. (A) Immunoblots showing the levels of NaK-β_1_ and NaK-α_1_ in Caki-1 cells stably transfected with scrambled shRNA (SCRAM) or shRNA against NaK-β_1_ (shRNA-β). Two clones (cl#1 and cl#2) were further studied. Actin was used as a loading control. (B) Rubidium uptake assay showing Na,K-ATPase pump activity was comparable in SCRAM and shRNA-β cells. (C) Cells were treated with TRIAM, DEX, or FLUOR, and NaK-β_1_ and NaK-α_1_ expression was evaluated by immunoblotting.

Since GCs may mediate anti-tumor effects through multiple mechanisms, we determined whether the enhanced cell-cell aggregation and reduced invasion by these drugs is mediated through NaK-β_1_ up-regulation. We generated a NaK-β_1_ knockdown (shRNA-β) Caki-1 cell line that exhibited 80–90% reduction in NaK-β_1_ (Fig 6A) [18, 24]. Strikingly, the level of NaK-α_1_ and the pump activity did not change significantly in these cell lines (Fig 6B). Furthermore, we tested the ability of the compounds to restore NaK-β_1_ expression in the knockdown clones. All three GCs induced NaK-β_1_ expression in shRNA-β clones, but expression levels remained below that present in parental or scrambled shRNA transfected cells (Fig 6C).

**Fig 7:**
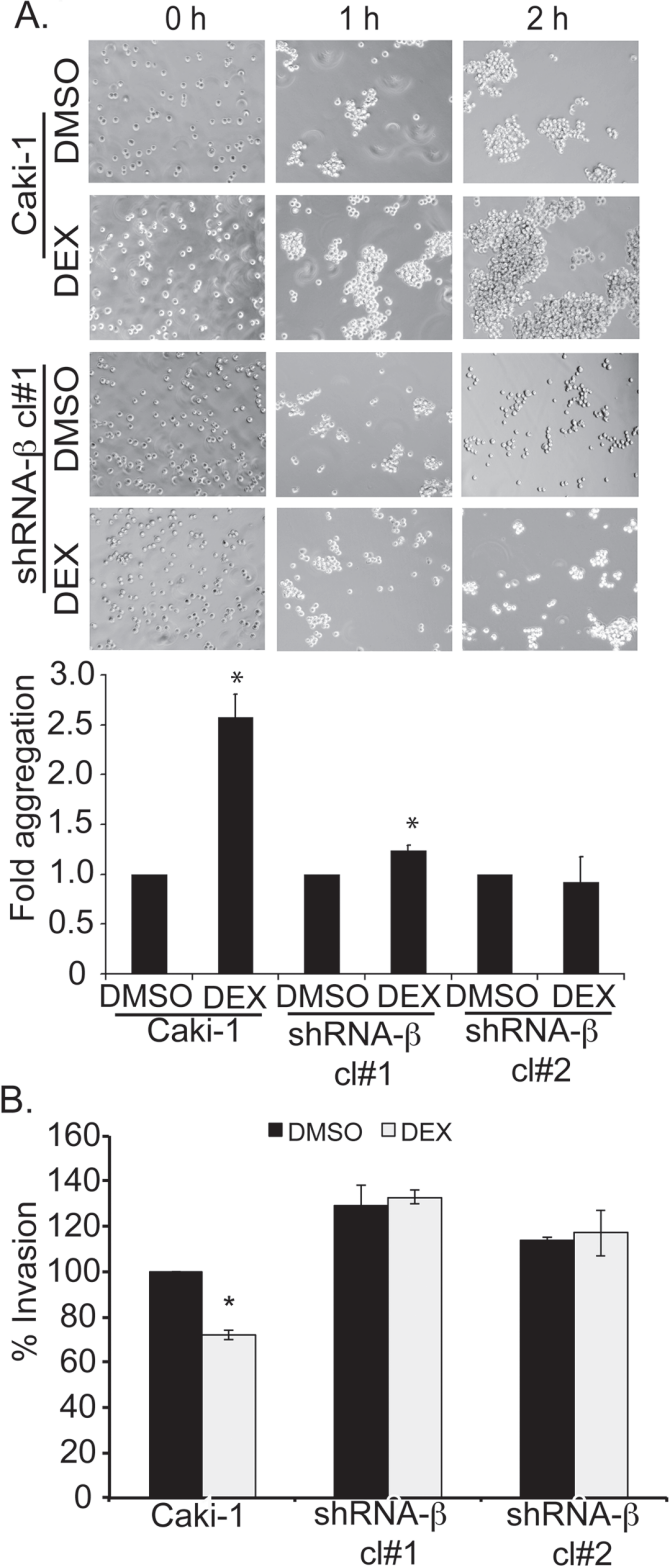
GCs are less effective on NaK-β_1_ knockdown cells. (A) Cells were pre-treated with DEX and cell aggregation was assessed. Phase contrast images were acquired to depict the extent of cell-aggregation. The extent of cell-cell aggregation was quantified at 1 hr post-shaking and DEX treated conditions were expressed as a fold of DMSO treated samples. Asterisk indicates p-value was < 0.05. (B) Transwell invasion assay showing the relative invasiveness of Caki-1 and shRNA-β cells in the presence or absence of DEX pre-treatment. The invasion exhibited by DMSO was considered 100% for each cell line. The graph represents the mean±SE from three independent experiments (p-value < 0.05).

A cell-cell aggregation assay revealed that DEX-treated Caki-1 cells produced larger cell aggregates compared to vehicle-treated cells. The shRNA-β knockdown cells were mostly mono-dispersed with occasional clusters at 2 hr (Fig 7A). DEX did not induce aggregation in either clone of shRNA-β cells (Fig 7A).

To assess whether GCs mediate anti-invasive effects through NaK-β_1_, we determined the extent of cell invasion through collagen-coated filters in Caki-1 parental and shRNA-β cells. In the presence of 100 nM DEX, the invasion of Caki-1 cells was reduced to 75±1.7% of DMSO-treated cells (Fig 7B). In contrast, shRNA-β cells, in which DEX was not effective at up-regulating NaK-β_1_ expression, displayed no reduction in invasion (Fig 7B). We have shown previously that cell invasiveness was inversely proportional to NaK-β_1_ levels [14, 22]. As expected, untreated shRNA-β cells were more invasive than the parental cells. Taken together, these results demonstrate that GCs mediate suppression of invasiveness through the up-regulation of NaK-β_1_.

### GCs induce MET like phenotype in Caki-1 cells

**Fig 8:**
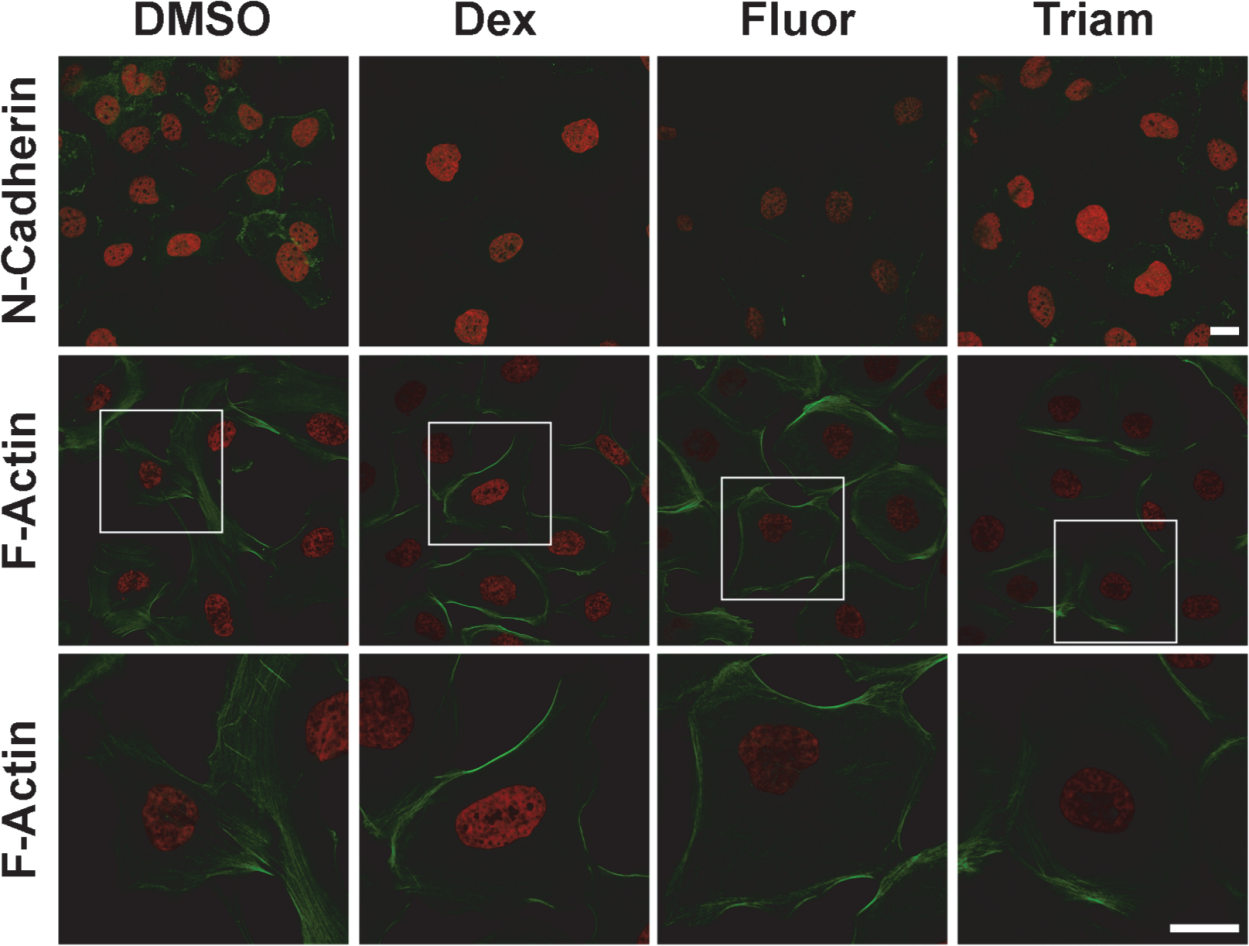
GCs promoted mesenchymal-to-epithelial transition of kidney cancer cells. Representative images of Caki-1 cells treated with GCs. Serum starved cells were treated with 10 μM GCs for three days and then immunostained. Staining of N-Cadherin (green) is shown with DAPI (red). F-Actin was stained with Alexa 488-conjugated phalloidin along with DAPI. Magnified images of the regions marked by white boxes are shown at the bottom. Scale bars represent 20 μm.

We have shown earlier that NaK-β_1_ expression is reduced during TGF-β induced EMT [18]. Carcinoma cell lines which have lost their epithelial phenotype also revealed reduced levels of NaK-β_1_ [9]. In addition, knockdown of NaK-β_1_ in Caki-1 cells induced mesenchymal phenotype which was accompanied by enhanced expression of fibronectin and smooth muscle actin which are markers upregulated during EMT [18]. Since NaK-β_1_ levels are inversely correlated with the acquisition of EMT, we reasoned that upregulated NaK-β_1_ expression by GCs might suppress the EMT phenotype. Consistent with this notion, GC treated cells showed enhanced cell to cell contact and revealed a more compact epithelial phenotype (Fig 8). Immunofluorescence analysis of N-cadherin, a mesenchymal marker which is upregulated during EMT was reduced by GC treatment. Invasive carcinoma cells show extensive stress fibers which is essential for their invasiveness and motility. GC treated Caki-1 cells showed drastically reduced stress fibers and increased cortical actin networks (Fig 8). These results suggest that GCs induce MET like phenotype in RCC cells.

### GCs suppress tumor growth and invasiveness in vivo

**Fig 9:**
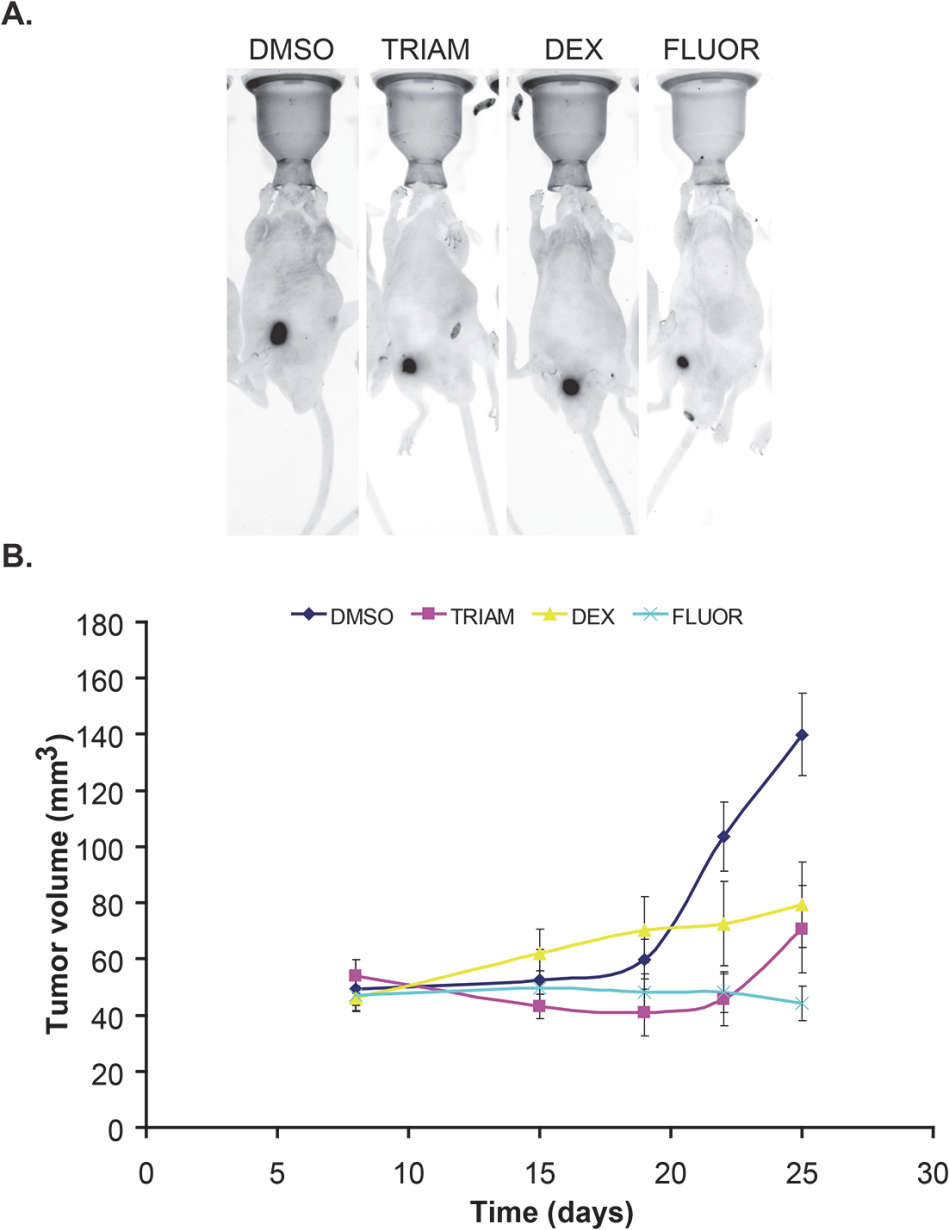
GCs suppress growth in a subcutaneous xenograft mouse model. (A) Representative images of tumor-bearing mice treated with DMSO or GCs and acquired by Kodak in vivo multi-spectral imaging system are shown. (B) Tumor volume estimated by caliper measurements plotted as a function of time. Error bars denote SE of the mean (n = 5 per each group).

To study the effect of GCs on RCC tumor growth, Caki-1 cells expressing ptd-Tomato Red were generated and subcutaneously injected into SCID mice. Tumor growth was monitored by *in vivo* imaging and calipers. After 25 days, tumor volumes were strikingly smaller in the mice administered with any of the three drugs compared to vehicle treatment (Fig 9A and 9B). TRIAM and DEX slowed tumor growth, but did not completely prevent tumor growth over the 25 day period (Fig 9B). In contrast, FLUOR-treated mice displayed minimal tumor growth. On day 25, the tumor volume in TRIAM, DEX, and FLUOR-treated mice was smaller than the control (DMSO) group (70.6±15.7 mm^3^ (p-value = 0.20), 79.2±15.2 mm^3^ (p-value = 0.22), 44.2±6.0 mm^3^ (p-value = 0.02), respectively, compared to 140.0±14.7 mm^3^ for DMSO). Only FLUOR-treated mice showed a statistically significant effect in limiting tumor growth.

**Fig 10:**
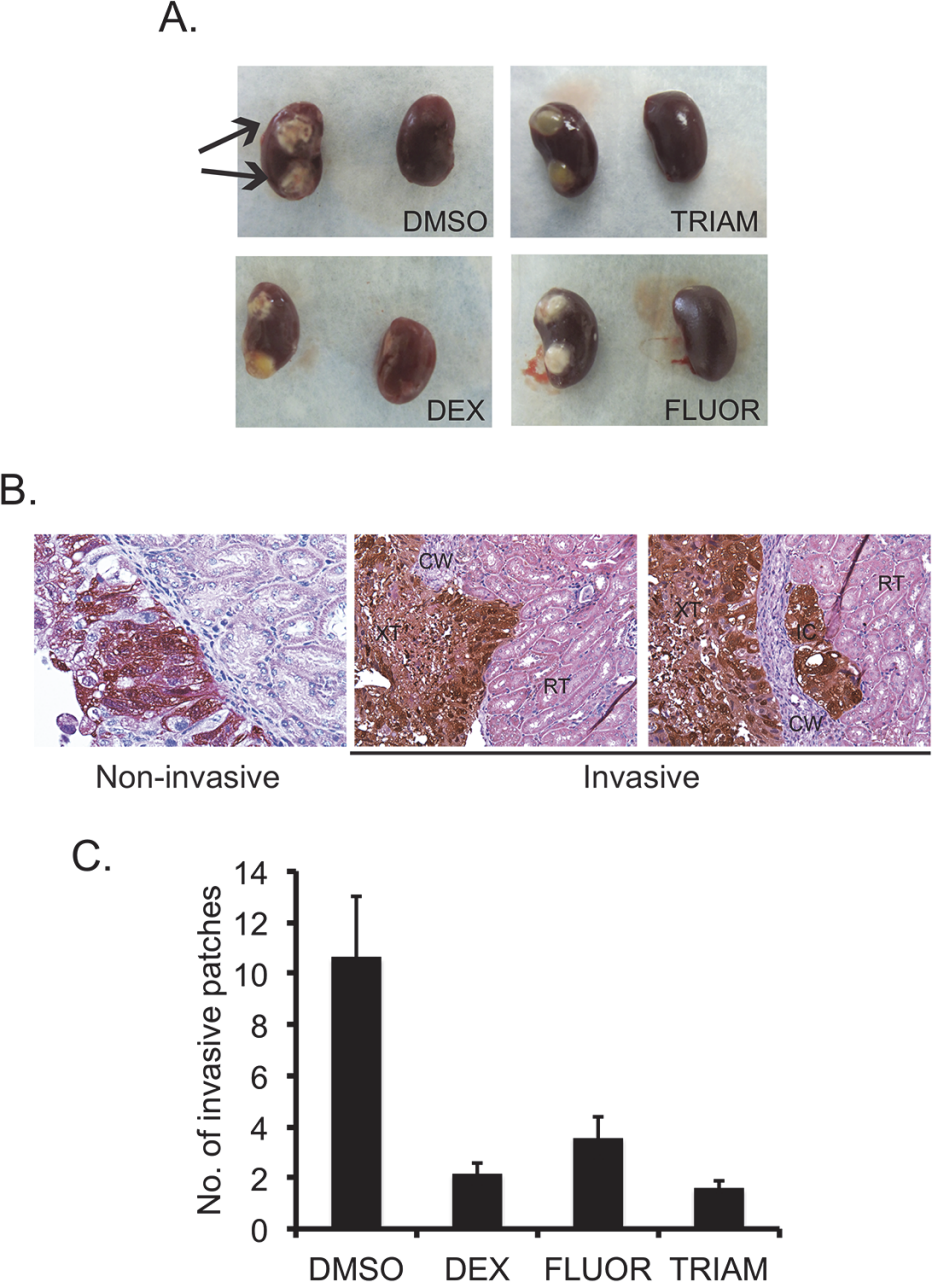
GCs reduce local invasiveness in an orthotopic renal xenograft mouse model. (A) Representative images of the left (with tumor implants) and right (normal) kidneys harvested from tumor-bearing mice. Arrows show compact tumors in DMSO treated mice. (B) The tumor-bearing kidneys were paraffin-embedded and sectioned. The sections were stained for antibodies against DsRed to visualize tumor cells (brown). Representative images showing invasive (DMSO treated) and non-invasive (DEX treated) patches are shown. (C) The graph shows the average number of invasive patches. Error bars denote SE of the mean (n = 8). Magnification 600X

To test whether GCs reduce tumor invasiveness *in vivo*, Caki-1 cells harboring ptd-Tomato Red were embedded in collagen and orthotopically implanted into the sub-renal capsule of SCID mice. Mice were treated with 5 mg/kg of TRIAM, DEX, or FLUOR every other day and tumors were harvested after five weeks. GC-treated mice displayed tumors that were compact compared to DMSO-treated mice where the tumors were more dispersed (Fig 10A arrows). To monitor local invasiveness, we stained tumor cells in kidney sections using an antibody directed against DsRed and quantified the invasiveness. Regardless of treatment, invasion of tumor cells into the renal parenchyma was detected in mice from each group. However, DMSO-treated mice had the largest number of invasive patches where the invading cells migrated into the cortex and medullary regions (Fig 10B and 10C). TRIAM, DEX, and FLUOR-treated mice showed reduced number of invasive patches compared to DMSO (Fig 10C) (p-values for TRIAM = 0.0182, DEX = 0.005134, FLUOR = 0.027116). Taken together, these data show that TRIAM, DEX, and FLUOR limited tumor invasiveness *in vivo*.

## Discussion

The major clinical use of GCs in cancer therapy has largely been a supportive one to reduce the side effects associated with cytotoxic chemotherapy. Graft versus host disease (GVHD) is a common and occasionally life-threatening side effect of bone marrow and stem cell transplants in patients, and GCs are also used to control GVHD. Steroid medications are particularly helpful in people who have spinal cord compression pain and nerve pain, which is common in patients with multiple myeloma. While GCs are not usually used alone, they are very effective and often lower the need for other pain medications. In addition to their palliative role, GCs are part of the standard treatment regimen for hematological malignancies. Dexamethasone is also used to treat patients with hormone refractory prostate cancer that has metastasized. Despite these multiple uses of GCs in the clinic, the mechanism by which GCs control tumor growth is not known. In this study, for the first time, we provide mechanistic evidence that GCs suppress RCC tumor growth and invasion by up-regulating the expression of a cell-adhesion molecule, Na,K-ATPase β_1_-subunit and suppressing expression of molecular markers associated with EMT.

In our high-throughput screen, a majority of the hit compounds identified were GCs, suggesting these are key regulators of NaK-β_1_ expression. Regulation of NaK-β_1_ expression by GCs, including TRIAM and DEX, occurs through the glucocorticoid receptor and/or mineralcorticoid receptor, which bind to *cis* elements in the NaK-β_1_ promoter to stimulate expression [5, 38–41]. However, the effect of FLUOR on NaK-β_1_ expression has not been studied. In agreement with the transcriptional regulation of NaK-β_1_ by GCs, we observed stimulation of the NaK-β_1_ promoter activity and increased transcripts in a dose-dependent manner, which correlated with increased protein. Previous reports have indicated that corticosteroids induce synthesis of NaK-β_1_, but the ability of these compounds to enhance the anti-tumor effects of NaK-β_1_ expression has not been tested. This reflects the discovery of corticosteroid effects on NaK-β_1_ expression, which occurred well before the tumor suppressor functions of NaK-β_1_ were identified.

We show that GCs increased surface expression of NaK-β_1_ resulting in enhanced cell-cell adhesion and suppressed cell motility and invasion. These effects are likely specific to GC mediated up-regulation of NaK-β_1_ since shRNA-β cells treated with GCs showed less cell-cell adhesion and were more motile compared to untreated parental cells. If GCs induced the cellular effects through NaK-β_1_ independent mechanisms, then the GCs should have enhanced cell-cell adhesion and suppressed the invasiveness of the shRNA-β cells.

RCC cells express E-cadherin, a calcium-dependent cell adhesion molecule and an invasion suppressor in epithelial cells. GCs did not increase the expression levels of E-cadherin (data not shown). Moreover the cell adhesion and invasion assays were performed under calcium-free conditions and treatment of cells with trypsin cleaves the extracellular domain of E-cadherin. Therefore, the increased aggregation and reduced invasion observed in GC-treated cells are likely due to enhanced expression of NaK-β_1_.

We confirmed that the GCs enhanced cell adhesion and suppressed of invasion in two well-characterized RCC cell lines. Our *in vivo* data suggests that GCs reduce growth and invasiveness of RCC tumors. Although all three GCs suppressed growth of tumors, FLUOR was the most and DEX the least effective in growth suppression *in vivo*. DEX suppressed growth of OUR-10, a RCC cell line, both *in vivo* and *in vitro* [42]. However, in our study, GCs did not suppress growth of RCC cells *in vitro*. This might be attributed to the fact that *in vitro* cells are exposed to the drug for a few days, whereas treatment *in vivo* occurred over several weeks. The prolonged and sustained exposure may be required to see inhibitory effects on tumor growth. It’s also possible that mechanisms which modulate growth regulation by GCs *in vitro* and *in vivo* are distinct. GCs may alter the tumor microenvironment *in vivo* to decrease tumor size. Using three different renal cell carcinoma cell lines, one group showed that treatment of cells *in vitro* and *in vivo* inhibited tumor growth through a possible mechanism involving inhibition of NF-kB and expression of its downstream products interleukin-6 (IL-6), interleukin-8 (IL-8), and vascular endothelial growth factor (VEGF) [40]. Since GCs bind to the glucocorticoid receptor, a transcriptional activator and repressor with multiple well-known targets, we cannot rule out the possibility that the observed anti-tumor effects are due solely to up-regulation of Na,K-β_1_. We did not measure levels of IL-6, IL-8, or VEGF, but we expect that they would be similarly decreased in tumor treated cells. As Arai *et al*. mentioned in their study, this may be a reason why we also did not observe a reduction of growth *in vitro*, but we did see dramatic changes *in vivo* [40]. In particular, the loss of IL-8 and VEGF would decrease tumor growth by limiting angiogenesis and neovascularization *in vivo*.

Our sub renal capsule xenograft model facilitated study of local invasiveness *in vivo*. Invasion was most effectively suppressed by TRIAM, whereas FLUOR was the least effective and DEX was intermediate. This result suggests that mechanisms which modulate *in vivo* growth and invasive properties of tumor cells are different. Future experiments are necessary to understand these mechanisms. These *in vitro* and *in vivo* observations demonstrate the proof of principle that elevation of NaK-β_1_ with GCs attenuates RCC tumor progression.

We showed earlier that an ovarian carcinoma sub-line (A2780/C10B) resistant to oxaliplatin has significantly reduced levels of NaK-β_1_ compared to non-resistant lines. Re-expression of NaK-β_1_ into this sub-line enhanced uptake of oxaliplatin and restored the sensitivity of these cells to oxaliplatin [43]. In this adjuvant setting, GC-stimulated up-regulation of NaK-β_1_ may play a dual role in suppressing tumor progression and enhancing the uptake of cytotoxic therapies. Combination therapies may be an interesting avenue to pursue, since the GCs in our study had a minimal oncolytic effect, which is in agreement with several published reports [44, 45]. Several reports have already noted GCs can potentiate or enhance the therapeutic effects of cisplatin and gemcitabine in different cancers, though the mechanism is not completely understood [46, 47]. It is plausible that the enhanced drug uptake is due to augmentation of NaK-β_1_. Future studies will address these possibilities.

The potential value of the steroids identified in the HTS in the setting of metastatic RCC has not been adequately studied. To our knowledge, the use of FLUOR and TRIAM in the treatment of renal cell carcinoma has been unexplored. In contrast, there have been a few case reports on the use of DEX in treatment of RCC. For example, it was shown that a patient diagnosed with poorly differentiated RCC showed a significant clinical response to DEX monotherapy. The patient did not receive any other systemic treatment or medication, but yet showed a size reduction of the primary kidney tumor along with a reduction in metastatic deposits in the lung, liver, and spleen [47]. There have been other case studies showing partial or complete regression of RCC when using other steroids identified in the HTS. For example, a group in Japan reported the complete regression of metastatic renal cell carcinoma following betamethasone treatment [48]. Another case study reported a patient with a 10-year complete remission of metastatic renal cell carcinoma undergoing prednisone monotherapy [46]. A recent study demonstrated that GCs suppress the invasion and migration of malignant glioblastoma cells *in vitro* [48]. GCs can also inhibit TGF-β1 induced EMT in lung epithelial cells [49] and in peritoneal mesothelial cells [50]. Here, we showed that treatment of GCs not only up-regulated NaK-β_1_, but also downregulated N-cadherin a marker up-regulated during EMT. In addition, actin stress fibers which are involved in the invasiveness and motility of carcinoma cells are drastically reduced in GC treated cells. These results are consistent with the notion that GCs induce MET like phenomenon and suppress EMT. It is possible that acquisition of MET like feature might have contributed to reduced tumor growth and invasiveness observed *in vivo*. Our data suggests a need for a re-examination of the use of GCs for the treatment of malignancies as it pertains to patients lacking NaK-β_1_ expression in late stages of disease. Future clinical trials are essential to validate the specific use of GCs in RCC treatments and the significance of enhanced NaK-β_1_ expression in the suppression of carcinoma progression.

## Supporting Information

S1 Table Steroidal activators. EC_50_ and fold activation values for each compound were determined from a single dose-response curve. Activation at each concentration was determined from three data points.
(TIF)
