# Supplementary figures and images for: Glucocorticoids Suppress Renal Cell Carcinoma Progression by Enhancing Na,K-ATPase Beta-1 Subunit Expression
Source: PLoS One. 2015 Apr 2;10(4):e0122442. doi: 10.1371/journal.pone.0122442 (PMC4383530; doi:10.1371/journal.pone.0122442)

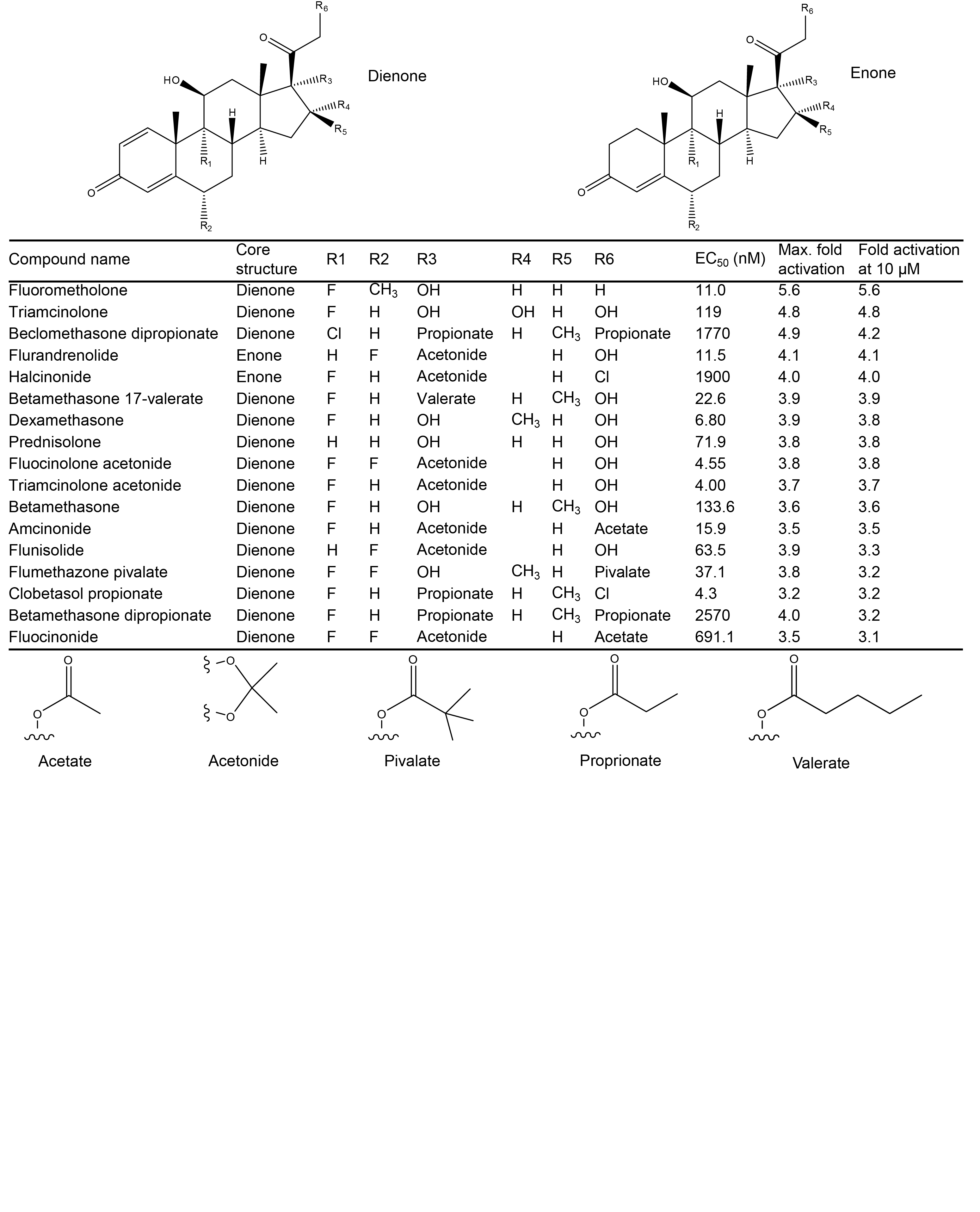

Supplement: S1 Table — EC50 and fold activation values for each compound were determined from a single dose-response curve. Activation at each concentration was determined from three data points. (TIF) [file pone.0122442.s001.tif]
